# Identification of Genetic Differentiation between Waxy and Common Maize by SNP Genotyping

**DOI:** 10.1371/journal.pone.0142585

**Published:** 2015-11-13

**Authors:** Derong Hao, Zhenliang Zhang, Yujing Cheng, Guoqing Chen, Huhua Lu, Yuxiang Mao, Mingliang Shi, Xiaolan Huang, Guangfei Zhou, Lin Xue

**Affiliations:** Nantong Key Laboratory for Exploitation of Crop Genetic Resources and Molecular Breeding, Jiangsu Yanjiang Institute of Agricultural Sciences, Nantong, China; National Institute of Plant Genome Research (NIPGR), INDIA

## Abstract

Waxy maize (*Zea mays* L. var. *ceratina*) is an important vegetable and economic crop that is thought to have originated from cultivated flint maize and most recently underwent divergence from common maize. In this study, a total of 110 waxy and 110 common maize inbred lines were genotyped with 3072 SNPs to evaluate the genetic diversity, population structure, and linkage disequilibrium decay as well as identify putative loci that are under positive selection. The results revealed abundant genetic diversity in the studied panel and that genetic diversity was much higher in common than in waxy maize germplasms. Principal coordinate analysis and neighbor-joining cluster analysis consistently classified the 220 accessions into two major groups and a mixed group with mixed ancestry. Subpopulation structure in both waxy and common maize sets were associated with the germplasm origin and corresponding heterotic groups. The LD decay distance (1500–2000 kb) in waxy maize was lower than that in common maize. Fourteen candidate loci were identified as under positive selection between waxy and common maize at the 99% confidence level. The information from this study can assist waxy maize breeders by enhancing parental line selection and breeding program design.

## Introduction

Waxy maize (*Zea mays* L. var. *ceratina*) is a type of maize with nearly 100% amylopectin in endosperm, which is mainly consumed as fresh vegetable in Asian countries such as in China, Japan, Korea, and the Philippines, and it is also used as raw material for food industries, textiles, adhesive, and paper industries [1–5]. Waxy maize was first discovered in China in 1908 [6] and was later found in other places in Asia [7, 8]. Waxy maize landraces are abundant in China, most of which are distributed in Southwestern China [2]. Chinese waxy maize is thought to have evolved from the non-glutinous domesticated American maize, which was introduced into China about 500 years ago [9]. Based on morphology, karyotype, isozyme, and DNA markers, waxy maize was suggested to have originated from the Yunnan-Guangxi region in China [10–13].

Interpreting genetic diversity in economically important crops allows breeders to consider using these trait reservoirs and, eventually, identify novel alleles or haplotypes to improve yield, quality, adaptation, and stress resistance [14, 15]. Understanding how genetic variation is distributed within and among different germplasm collections is vital to germplasm management by monitoring genetic shifts that occur during domestication, germplasm conservation, and breeding [16, 17]. Recently, application of RAPD and SSR markers has enabled identification of genetic diversity in waxy maize landraces and inbred lines; there is abundant genetic diversity in waxy maize, and waxy and common maize genetically differed [2, 18–22]. With the development of molecular systematics, comparison of DNA sequence variation between closely related species has provided insight into the amount of divergence between sibling species [1]. Sequence comparison and phylogenetic analysis for genes of *waxy* and *Globulin-1* indicated that Chinese waxy maize originated from cultivated flint maize and most recently diverged from common maize [1, 2].

The detection of genome-wide genetic diversity and identification of loci that contribute to domestication and improvement are essential for breeding superior varieties [23]. Genomic regions or genes affected by natural and artificial selection have been detected by studies in some plants [24, 25]. To date, genetic diversity and evolution analysis of waxy maize has primarily relied on analysis of small panels of accessions characterized by a limited number of markers or only some particular gene regions (e.g., *waxy* and *Globulin-1* gene) [1, 2, 11, 18, 26]. Single nucleotide polymorphism (SNP) genotyping is cost-effective, accurate, and fast; it has been frequently used for high-throughput analysis of plants, which facilitates genome-wide analysis of sequence variation, genetic diversity, and detection of outlier loci with selective signatures that affected genetic differentiation in germplasm collections [15, 27].

In this study, a wide array of China waxy maize and common maize inbred lines were genotyped with 3072 SNPs to analyze whether environment- and human-driven selection influenced the distribution of genetic variation among waxy and common maize; elucidate the genetic diversity present in a diverse set of germplasm accessions that have been used for different breeding objectives in modern plant breeding; and identify genomic regions that were potentially subjected to selection events using an *F*st outlier approach.

## Materials and Methods

### Plant materials

A total of 220 germplasm accessions were used to analyze the genetic differentiation between waxy and common maize in this study (S1 Fig, S1 Table). These accessions included 110 Chinese waxy maize accessions selected from a wide range of geographical locations in China to try to encompass genetic diversity within landraces and inbred lines and 110 common maize accessions composed of the traditional landraces and improved maize inbred lines. These accessions were assembled from the Jiangsu Maize Germplasm Bank in China.

### SNP genotyping

Total genomic DNA was extracted from young leaves of each of the genotypes using a modified CTAB method [28]. All 220 germplasm accessions in the studied panel were genotyped with the Maize SNP3K Beadchip (Illumina, San Diego, CA, USA) via the GoldenGate assay at the National Maize Improvement Centre of China, China Agricultural University. This SNP3K Beadchip contained 3072 random, good quality SNPs for genotyping, and the SNPs evenly covered the maize genome (including 1884 intragenic SNPs and 1188 intergenic SNPs) [29].

### Genetic diversity and genetic structure analysis

PowerMarker 3.25 [30] was used to evaluate the genetic diversity characteristics for each locus in the studied panel, including number of alleles, minor allele frequency (MAF), gene diversity, and polymorphism information content (PIC). In addition, to investigate the genetic distances between waxy and common maize, principal coordinate analysis (PCoA) and neighbor-joining cluster analysis based on Nei’s distance matrix [31] were conducted using GenAlex 6.5 [32] and PowerMarker 3.25 [30]. Analysis of molecular variance (AMOVA) was also performed to investigate the population difference within and among waxy and common maize germplasms using Arlequin 3.01 [33].

### Linkage disequilibrium (LD) analysis

The LD parameter (*r* ^*2*^) was calculated to estimate the degree of LD at *P* = 0.01 between pairwise SNPs for the entire dataset as well as separately for the waxy and common germplasm datasets using TASSEL 4.0 [34]; the cut-off value for *r* ^*2*^ was determined with a threshold of 0.1, as previously described [35]. To estimate the LD of the overall genome, the *r* ^*2*^ value was calculated for individual chromosomes using SNPs from the corresponding chromosome [23].

### Signatures of selection

Based on an *F*st-outlier detection method [36], LOSITAN [37] was used to identify the candidate loci under positive selection between waxy and common maize datasets. One hundred simulations with 1000 iterations were run for each pairwise comparison using the ‘neutral mean *F*st’ and ‘force mean *F*st’ options to increase estimate reliability. For the mutation model, an infinite alleles model was used. SNPs with *F*st values in the 99th percentile of neutral distribution were considered candidates for positive selection [24, 38].

## Results

### Genetic diversity

**Fig 1:**
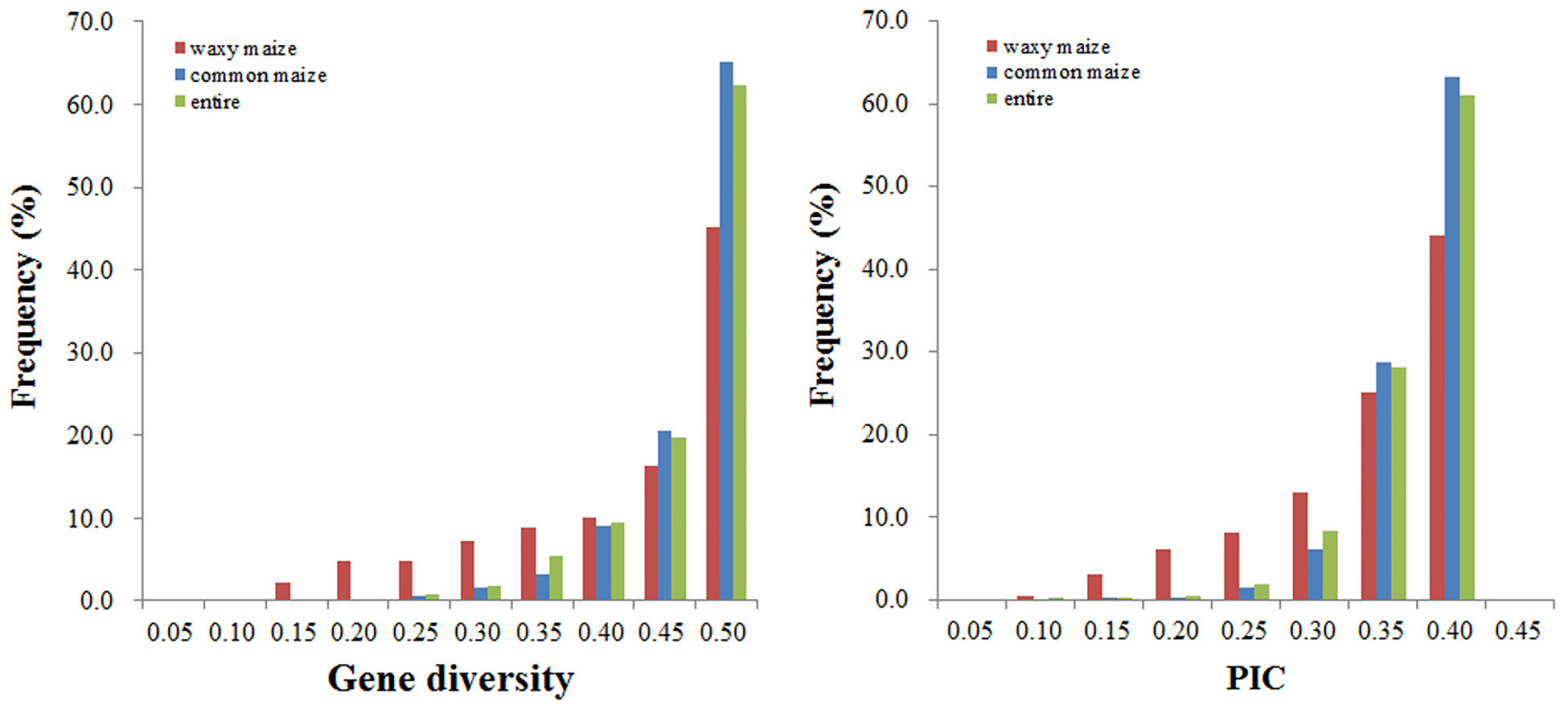
Frequency distribution of genetic diversity of gene diversity and PIC within the waxy maize, common maize and the entire datasets.

Initially, a total of 220 germplasm accessions in the studied panel that contained 110 waxy maize and 110 common maize germplasms were genotyped with 3072 SNPs. Genetic diversity analysis using PowerMarker 3.25 revealed SNPs with MAF ≥ 5% and missing data ≤ 20%, which were used for subsequent analysis. Based on these criteria, the genotypic dataset of all accessions was reduced to 2811 SNPs, whereas the waxy and common maize datasets were reduced to 2751 and 2835 SNPs, respectively. The diversity among waxy maize, common maize, and entire datasets revealed by the three different sets of SNP greatly varied (Table 1, Fig 1). For the waxy maize set, the average gene diversity was 0.398 with a rang of 0.095–0.500, and the PIC averaged 0.314, ranging from 0.091 to 0.375. The genetic diversity within the waxy maize germplasms was much lower than that in the common maize germplasms (Fig 1), which indicates that waxy maize had substantially lower genetic variability than common maize. For the entire dataset, the gene diversity indices and PIC values were 0.448 and 0.346, respectively.

**Table 1:** Gene diversity and PIC in the waxy maize, common maize and the entire datasets.

Datasets	Number of SNPs	Alleles per locus	Gene diversity	PIC
waxy maize set	2751	2.00	0.398	0.314
common maize set	2835	2.00	0.453	0.347
entire set	2811	2.00	0.448	0.346

### Population structure

**Fig 2:**
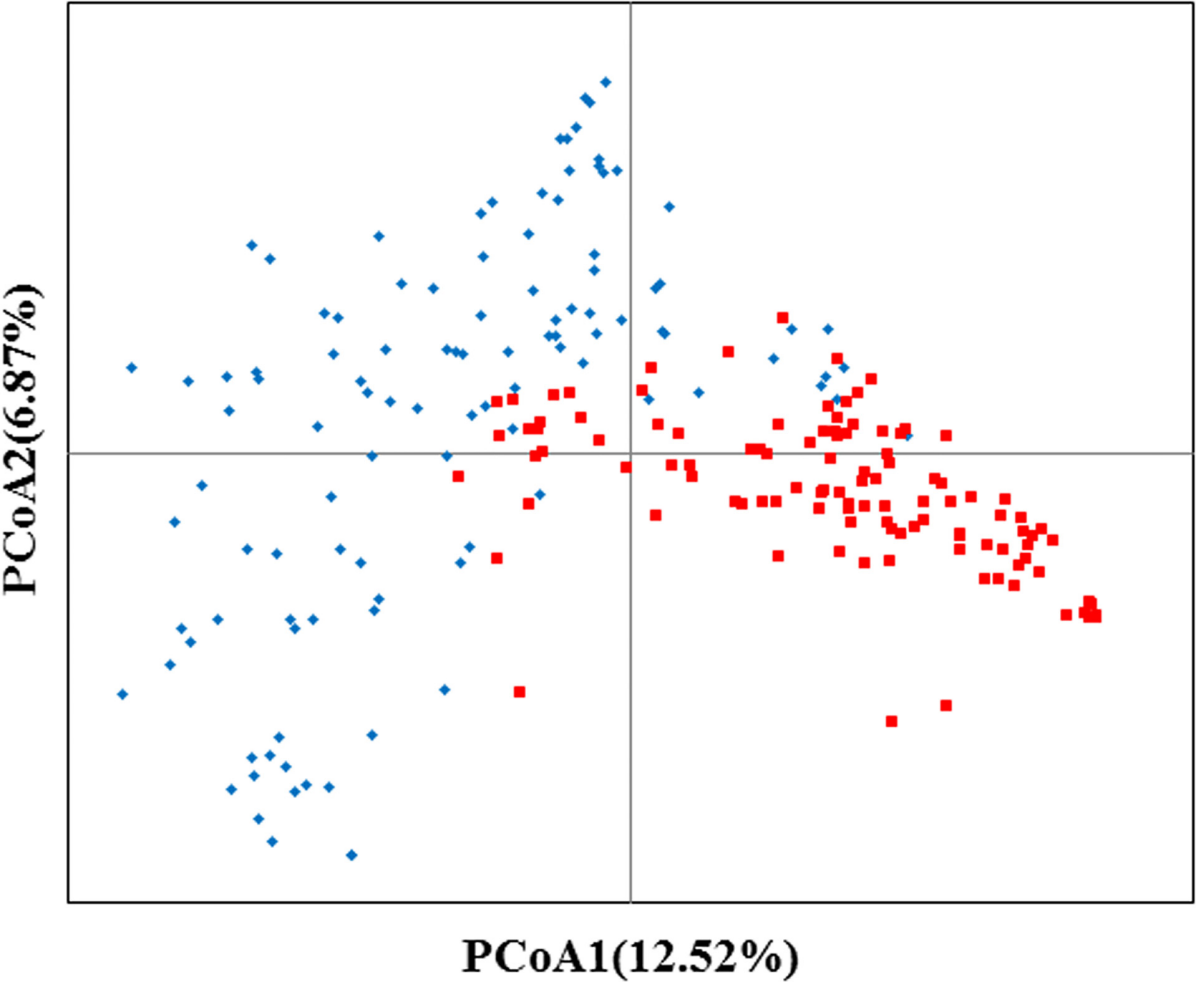
Plot of the first two PCoA axes using the 2811 SNPs. The red squares correspond to waxy maize germplasms; the blue rhombuses correspond to common maize germplasms.

A two-dimensional scatter plot that included all 220 accessions revealed that the first two principal components (PCoA1 and PCoA2) explained 12.52 and 6.87%, respectively, of the genetic variation among the studied groups (Fig 2). On the first axis, the majority of the waxy maize germplasms were separated from the common maize, though with some overlap. The PCoA data suggested that the tested germplasms might be grouped into two major groups (waxy group and common group) and a mixed group (mainly including some germplasms with mixed ancestry). The scatter plots of PCoA 1 vs PCoA 3 and PCoA 2 vs PCoA 3 also revealed similar results (S2 and S3 Figs).

**Fig 3:**
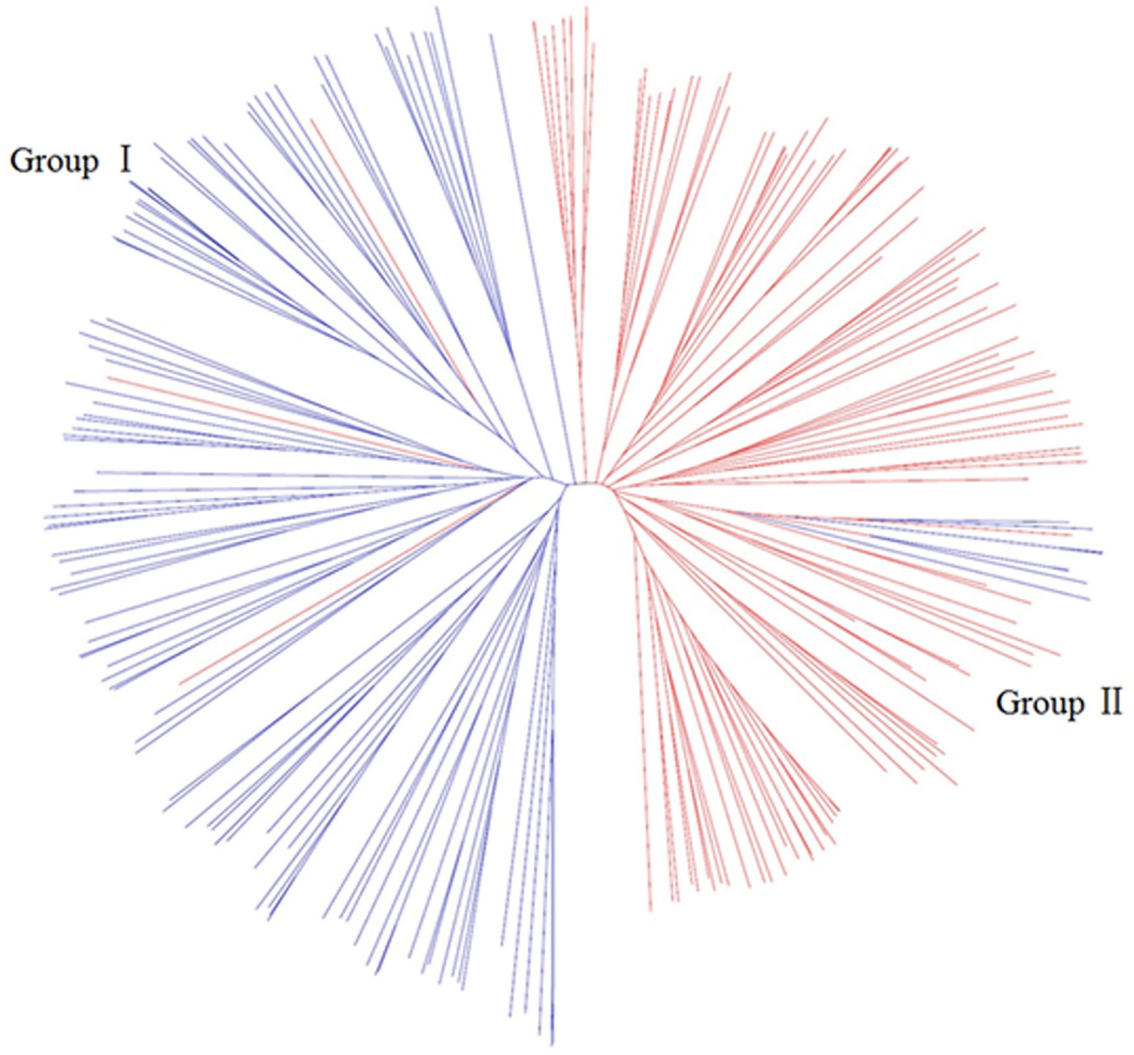
Neighbor-joining tree of all 220 germplasms inferred from 2811 SNPs.

The neighbor-joining tree (Fig 3) was consistent with the PCoA results. The 220 accessions clustered into two major groups, with several germplasms with mixed genetic background. Group I included 102 common maize germplasms and three waxy maize germplasms, whereas group II was composed of 107 waxy maize germplasms and eight common maize germplasms. In order to gain further insight into the population structure within waxy and common maize, neighbor-joining trees based on Nei’s genetic distance were constructed in waxy and common maize, respectively. The waxy and common maize germplasms both clustered into three subpopulations (S4 and S5 Figs). For the waxy maize, the germplasms in subpopulation P1 (including 33 germplasms) were mainly from Southeast China, derived from a core germplasm of ‘Tongxi 5’ in Southeast China; the 28 gremplasms in subpopulation P2 were mainly from Southwest China and Thailand with subtropical and tropical genetic background. While the last subpopulation P3 of waxy maize included 49 germplasms, mainly derived from another core germplasm of ‘Hengbai 522’ from North China. For the common maize, the first subpopulation P1 included 66 germplasms, mostly belonging to the ‘PB’ (derived from modern US hybrids); the subpopulation of P2 contained nine germplasms, with the genetic background of ‘Sipingtou’; the germplasms in the subpopulation P3 (including 35 germplasms) mostly belonged to the group of ‘Lancaster’.

The distribution of genetic variation within and among the waxy and common maize groups was estimated by AMOVA (Table 2). The results of AMOVA indicated that 9.54% of the total genetic variation was among the waxy and common maize groups, whereas 90.46% was among individuals within the waxy and common maize groups. In the waxy maize group, 14.89% of the total genetic variation was among subpopulations, and 85.11% was among individuals within the subpopulations. The AMOVA also revealed that 11.45% and 88.55 of the total genetic variation was among and within the subpopulations in the common maize group, respectively.

**Table 2:** AMOVA and *F*_*ST*_ for the studied waxy and common maize groups.

Dataset	Source of variation	*d*.*f*	Sum of squares	Variance components	Percentage variation	*P*-value
Entire set	Among groups	1	13466.56	58.68	9.54	< 0.001
	Within groups	438	243634.70	556.24	90.46	< 0.001
	Total	439	257101.26	614.93		
	Population pairwise *F*_*ST*_: 0.095 (*P* < 0.001)					
Waxy maize	Among subpopulations	2	12221.49	79.50	14.89	< 0.001
	Within subpopulations	217	98637.68	454.55	85.11	< 0.001
	Total	219	110859.18	534.05		
	Population pairwise *F*_*ST*_: 0.149 (*P* < 0.001)					
Common maize	Among subpopulations	2	8533.93	74.59	11.45	< 0.001
	Within subpopulations	217	125201.39	576.97	88.55	< 0.001
	Total	219	133735.32	651.55		
	Population pairwise *F*_*ST*_: 0.114 (*P* < 0.001)					

### LD

LD between pairwise SNPs across the entire genome was investigated among waxy maize, common maize, and entire datasets (Table 3). In the 220 accessions, the LD of 36.32% of the pairwise SNPs was significant across 10 chromosomes at the 0.01 level, 34.32% of which had *r* ^*2*^ > 0.1. For the waxy maize dataset, 25.16% of pairwise SNPs showed significant LD, 65.83% of which had *r* ^*2*^ > 0.1. Moreover, 32.07% of pairwise SNPs showed significant LD in the common maize dataset, 69.73% of which had *r* ^*2*^ > 0.1. The amount of LD distinctly differed across the 10 chromosomes (Table 3). For example, the percentage of pairwise SNPs in significant LD was 29.70% on chromosome 9 in waxy maize dataset, which was higher than that in other chromosomes, 72.28% of which had *r* ^*2*^ > 0.1.

**Table 3:** Percent of pairwise SNP markers in LD at *P* = 0.01 in the different maize datasets.

Chr.	SNPs			Pairwise SNPs in LD(%)			Pairwise SNPs in LD with *r* ^*2*^ > 0.1 (%)		
	entire	waxy maize	common maize	entire	waxy maize	common maize	entire	waxy maize	common maize
1	350	332	354	27.46	19.97	21.70	25.63	60.13	60.69
2	275	266	279	41.38	22.83	42.30	43.02	64.63	75.86
3	214	211	214	33.98	23.47	31.86	29.80	62.61	68.86
4	407	409	412	39.21	31.70	34.09	35.13	69.87	71.21
5	286	276	291	32.25	19.27	27.68	28.00	60.34	64.83
6	263	256	263	33.93	27.81	30.59	27.05	66.05	67.02
7	243	239	247	34.59	22.45	29.25	30.14	61.70	66.78
8	401	399	404	42.92	28.59	37.57	43.12	68.97	74.25
9	200	190	199	38.27	29.70	32.77	36.12	72.28	68.87
10	172	173	172	37.28	20.98	32.22	32.15	61.47	69.62
All	2811	2751	2835	36.32	25.16	32.07	34.32	65.83	69.73

**Fig 4:**
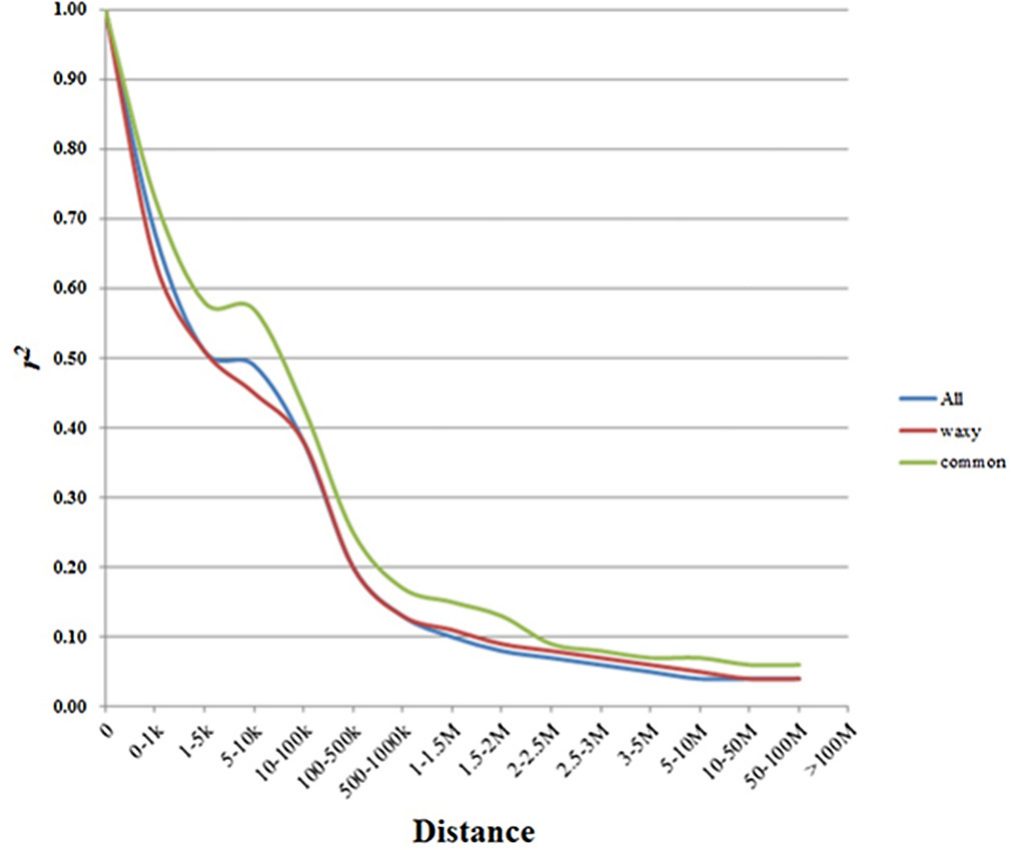
LD within chromosomes among different groups.

The average LD decay distance with *r* ^*2*^ > 0.1 varied across chromosomes among different groups, as shown in Fig 4. The LD decay distance in the waxy maize dataset was about 1500–2000 kb, which was lower than that in the common maize dataset (2000–2500 kb) but larger than that in the entire dataset (1000–1500 kb).

**Fig 5:**
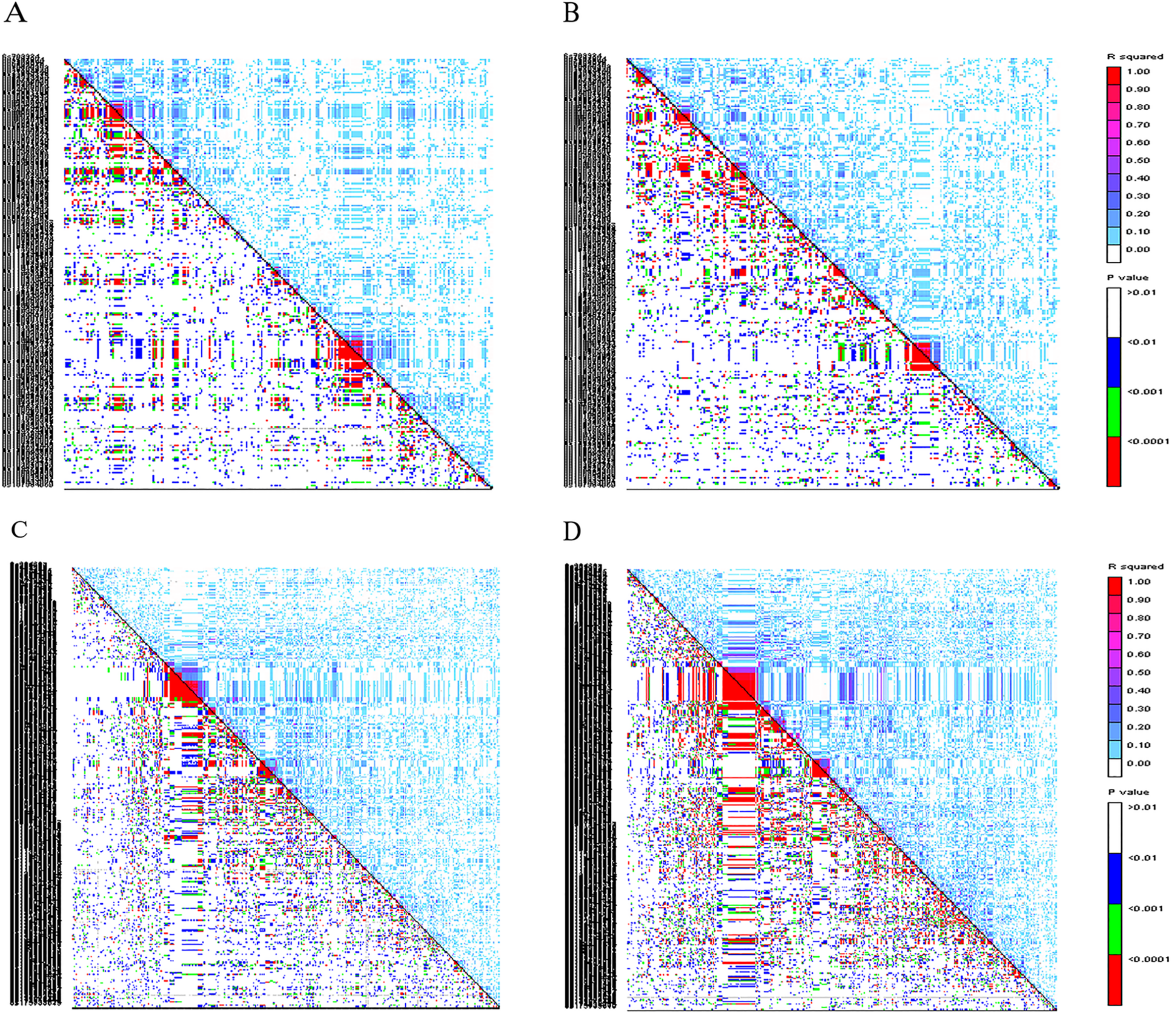
LD patterns on chromosomes 6 and 8 in waxy maize (A and C) and common maize (B and D) genotyped with 2751 and 2835 SNPs, respectively. *r* ^*2*^ for each pair of markers are presented in the upper triangle and their corresponding tests (*P* value) in the lower triangle.

For the waxy and common maize group individually, the extent of LD also greatly varied from region to region within different chromosomes (S1 File). Overall, the significant pairwise SNPs in LD within chromosomes in common maize distributed broader, and the LD degree was higher than those in waxy maize, except for chromosome 9. There were two significant big LD blocks on chromosomes 6 and 8 in the common maize (Fig 5, S2 Table). A distinct LD block with P < 0.0001 and R^2^ ≥ 0.9 was clearly shown on the chromosome 6 (Fig 5B), which contained 10 SNPs and spanned around 680 kb. Another big LD block with P < 0.0001 and R^2^ ≥ 0.9 was located on the chromosome 8, including 26 SNPs in a genomic region of 3.9Mb (Fig 5D).

### Candidate loci under positive selection

LOSITAN enabled detection of 14 SNP loci under positive selection that fell outside of the 99% confidence interval from pairwise comparisons between waxy and common maize datasets (Table 4). The *F*st values of these 14 loci ranged from 0.32 to 0.54, with an average of 0.45. The 14 SNP loci primarily located on seven chromosomes except for chromosome 4, 8, and 10. Among these loci, seven (50.0%) were localized on chromosome 9. The putative functions of the genes within which the outlier loci are located or the nearest genes from the outlier loci were inferred from the MaizeGDB database (www.maizegdb.org). The putative functions were mainly involved in plant growth and biotic or abiotic stress response, which are likely targets for modification through selection in breeding programs.

**Table 4:** Candidate loci under positive selection detected in this study.

SNP marker	*F*st	*P* (simul *F*st < sample *F*st)	Chr.	Position	Gene/Closest gene	Distance from the gene (kb)	Annotation	Putative functions
PZE-101001044	0.322	0.993	1	1968281	GRMZM2G060296	0.0	signal recognition particle receptor homolog1	encode enzymes in the lignin biosynthetic pathway [39]
SYN29562	0.431	0.992	2	236816133	GRMZM2G016236	0.0	hypothetical protein	
PZE-103035649	0.536	0.996	3	28922651	GRMZM5G891944	0.0	hypothetical protein	
PZE-103098628	0.410	0.992	3	158889049	GRMZM2G471529	6.4	histidine kinase2	regulate vascular tissue development [40]
PZE-105127960	0.373	0.995	5	185448519	GRMZM2G153227	0.0	H/ACA ribonucleoprotein	post-transcriptional RNA modification [41]
PZE-106059005	0.491	0.994	6	107885930	GRMZM2G392700	0.0	smr domain containing protein	chloroplast ATP synthase [42]
SYN24393	0.416	0.996	7	143410327	GRMZM2G147422	0.0	hypothetical protein	
PZE-109009763	0.540	1.000	9	10806295	GRMZM2G061257	2.6	leucine-rich repeat receptor-like protein kinase	signal response [43]
PZE-109023988	0.451	1.000	9	24141698	GRMZM2G122135	2.5	hypothetical protein	
PZE-109038023	0.468	1.000	9	55858508	GRMZM2G065694	2.3	EREBP-4 like protein	stress response [44]
PZE-109055211	0.374	0.995	9	95742546	GRMZM2G160730	3.2	AP2 domain containing protein	stress response [45]
PZE-109082403	0.507	0.992	9	130925219	GRMZM2G012970	6.9	hypothetical protein	
PZE-109089936	0.537	0.994	9	137787366	GRMZM2G078469	3.2	hypothetical protein	
PZE-109090207	0.417	0.992	9	138143188	GRMZM2G015730	5.9	alpha-taxilin	stress response [46]

## Discussion

Understanding genetic resources is a pivotal step to exploit the available information for breeding, especially if the resources are well adapted to local environments or has not been exposed to modern breeding [14, 15]. It is commonly thought that crops are bereft of genetic variation compared with their wild relatives [2]. In this study, genetic diversity information, including gene diversity index and PIC analysis, revealed substantial genetic diversity within each of the waxy and common maize groups, and that genetic variation in waxy maize was lower than that in common maize. The possible reason for the reduction of genetic diversity in waxy maize might be that waxy maize experienced a genetic bottleneck or founder effect during its improvement, especially during modern waxy maize breeding [2, 47]. The results of SNP-based diversity analysis between waxy and common maize were consistent with those of a previous study that used SSR markers [22], but also indicated a higher level of genetic differentiation between waxy and common maize than previously detected.

The population structure, extent of genetic differentiation, and relationship patterns were explored among the 220 accessions using PCoA and neighbor-joining cluster analysis. The two different multivariate methods supported the presence of the two genetically distinct groups (waxy and common maize) and a mixed group (mainly including a few germplasms with mixed ancestry, which indicates that introgression or gene flow occurred during modern cultivar breeding in waxy and common maize), which was overall relatively consistent with the pedigree information. The respective clustering result of waxy and common maize groups suggested that both groups might be clustered into three subpopulations, which was consistent with corresponding heterotic groups established based on the pedigree information and the experience of breeders on the combining ability of inbred lines in both waxy and common maize groups [27, 48]. The AMOVA detected higher differences among individuals within subpopulations than among subpopulations in both waxy and common maize groups, and whereas the proportion of genetic variation within subpopulations reflected high levels of genetic diversity [49].

LD levels varied both within and between different species [50]. In the present study, a total of 25.16% and 32.07% of the SNP pairs across chromosomes exhibited significant LD in the waxy and common maize sets, respectively. The average of LD decay distance in waxy maize (1500–2000 kb) was lower than that in common maize (2000–2500 kb). This difference may have occurred because waxy maize has experienced more intensive recombination, and contains more rare alleles (137 of 2751 SNPs with MAF < 10% in waxy maize, while 7 of 2835 SNPs with MAF < 10% in common maize in the this study, data not shown) than common maize through its domestication and breeding history [27]. The higher LD in common maize group could, also, be a result of population stratification [51]. The extent of LD also greatly varied from region to region within different chromosomes in both waxy and common maize. In common maize, two significant big LD blocks were clearly shown within the genomic regions of 680kb and 3.9Mb on chromosomes 6 and 8, respectively. In terms of putative functions of the genes identified in these regions, the most represented biological processes were related to responses to biotic and abiotic stresses[52–56], photosynthesis[57, 58], modulating development [59–61] and transposable element genes (S2 Table). These ‘hitchhiking’ might be footprints of more stringent artificial selection (domestication and breeding) for specific breeding targets in common maize than that in waxy maize, such as high yield, stress resistance, etc. [62]

In all of the examined datasets in this study, the extent of LD decay was greater than that in highly exotic germplasms (1–10 kb) [63]. Such long-range LD in our study is typical of breeding germplasms [29, 64, 65] and is expected in germplasms that experienced intensive inbreeding with limited opportunities for genetic recombination. This also most likely reflects a small number of founder lines as the source of the germplasm through recent breeding practices [29, 65].

Identification of loci that undergo positive selection is a fundamental step in understanding how populations adapted to specific environments and agronomic practices [15]. To date, the search for targets of domestication selection among waxy and common maize has primarily relied on the analysis of limited gene regions with a small amount of accessions [1, 4]. High-density SNP genotyping provides the opportunity to detect genomic regions affected by selective events [15]. In our study, 14 candidate loci under positive selection were identified across the entire genome using 3072 SNPs. Of these, six were located in genes, whereas eight were intergenic. The putative annotations of the potential causal genes, in which the candidate loci are located or are closest to, were inferred from the MaizeGDB database. A high proportion of these annotations are for genes involved in stress response, but caution should be taken when interpreting the results of outlier detection as a direct cause and effect [66]. Because of the higher LD in the studied panels, it is likely that the actual genes related to environment- and human-driven selection might be located in the genomic regions in LD where the detected candidate selection loci are located. The candidate loci identified in this study exhibited a disproportionate bias, with 42.86% located on chromosome 9. This result indicates that there might be ‘hot spots’ for directional selection with specific breeding targets (such as high yield, stress resistance, taste, and nutritional value).

For getting more meaningful information of genetic differentiation between waxy and common maize for our breeding programme, genome-wide analysis of genetic diversity, LD and signatures of selection were performed in this study. However, no LD block was identified in waxy region and other important genic regions, and only a set of 14 loci were identified under positive selection. These ascertainment bias might be caused by the sampling bias (using genetically narrow breeding lines) and using a pre-defined SNP chips mainly developed for common maize inbred lines[29, 67, 68]. In the further study, the assessment of genotypic data will be performed in a larger population size with more diverse genetic background, and large-scale non-preferential molecular markers will be used for better understanding the fixation of alleles as well as genetic draft during the selection pressure, getting more fixed regions under positive selection.

## Supporting Information

S1 Fig Figure of ears of waxy maize and common maize. A, ear of waxy maize; B, ear of common maize.
(TIF)

S2 Fig Scatter plot of PCoA1 vs PCoA3 using the 2811 SNPs. The red squares correspond to waxy maize germplasms; the blue rhombuses correspond to common maize germplasms.
(TIF)

S3 Fig Scatter plot of PCoA2 vs PCoA3 using the 2811 SNPs. The red squares correspond to waxy maize germplasms; the blue rhombuses correspond to common maize germplasms.
(TIF)

S4 Fig Neighbor-joining tree of waxy maize germplasms inferred from 2751 SNPs. (TIF)

S5 Fig Neighbor-joining tree of common maize germplasms inferred from 2835 SNPs. (TIF)

S1 Table List of the germplasm accessions used in the study. Information provided in this supplemental table includes inbred line or landrace name, and which group the line was assigned to (waxy maize and common maize).
(DOCX)

S2 Table Genes and SNPs identified within the distinct LD blocks on chromosome 6 and chromosome 8. (DOCX)

S1 File LD comparasion within all chromosomes between waxy and common maize. (PDF)
