# Supplementary figures and images for: Identification of Genetic Differentiation between Waxy and Common Maize by SNP Genotyping
Source: PLoS One. 2015 Nov 13;10(11):e0142585. doi: 10.1371/journal.pone.0142585 (PMC4643885; doi:10.1371/journal.pone.0142585)

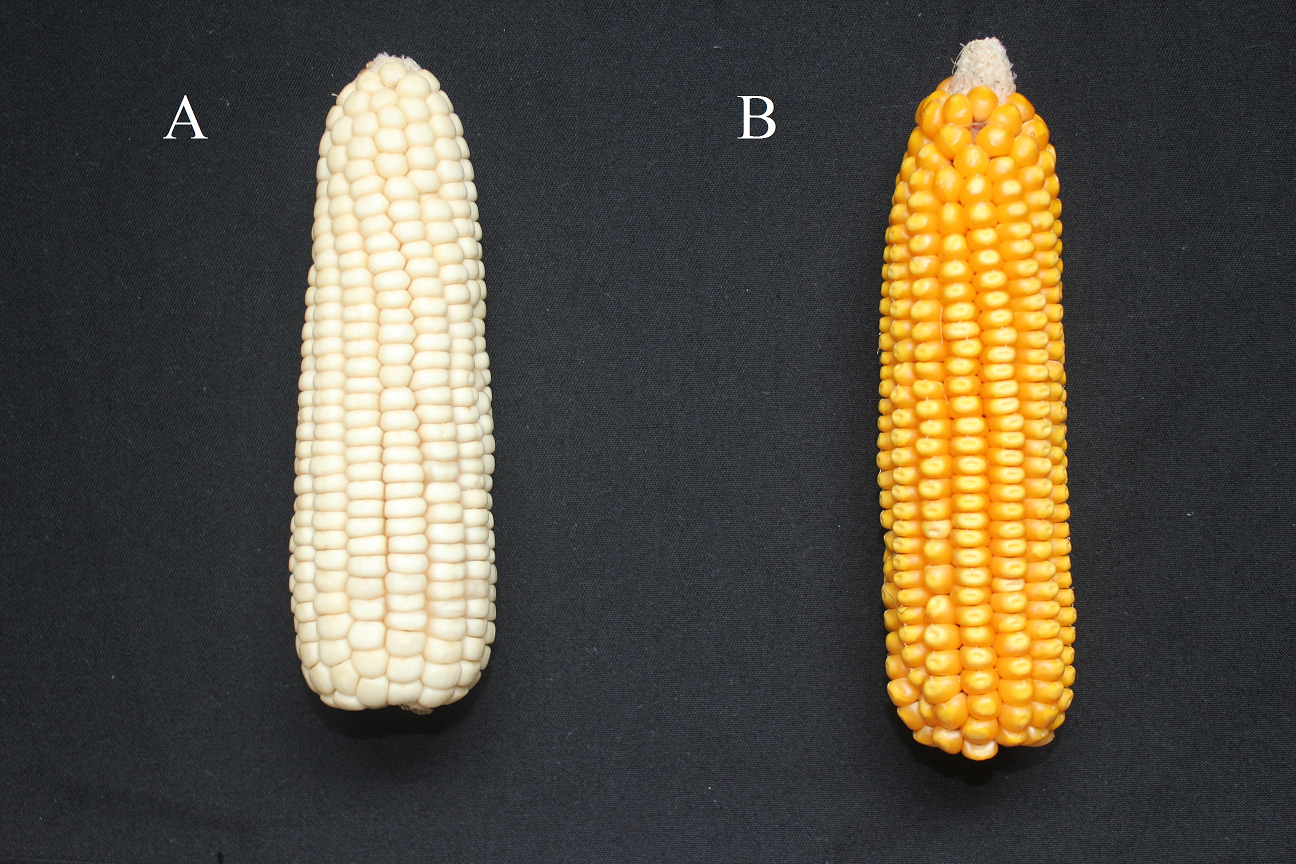

Supplement: S1 Fig — A, ear of waxy maize; B, ear of common maize. (TIF) [file pone.0142585.s001.tif]

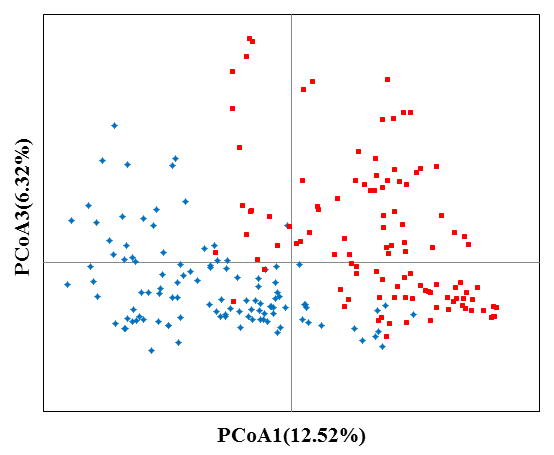

Supplement: S2 Fig — The red squares correspond to waxy maize germplasms; the blue rhombuses correspond to common maize germplasms. (TIF) [file pone.0142585.s002.tif]

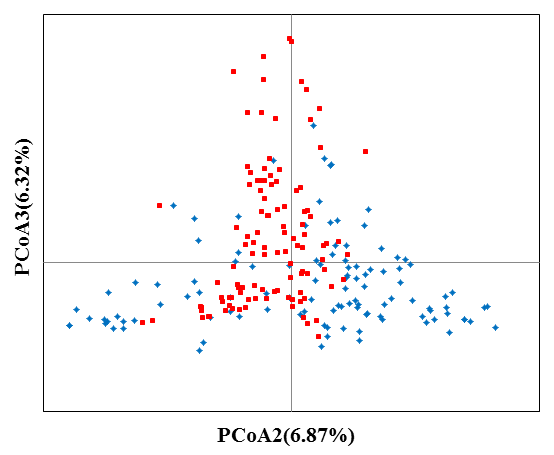

Supplement: S3 Fig — The red squares correspond to waxy maize germplasms; the blue rhombuses correspond to common maize germplasms. (TIF) [file pone.0142585.s003.tif]

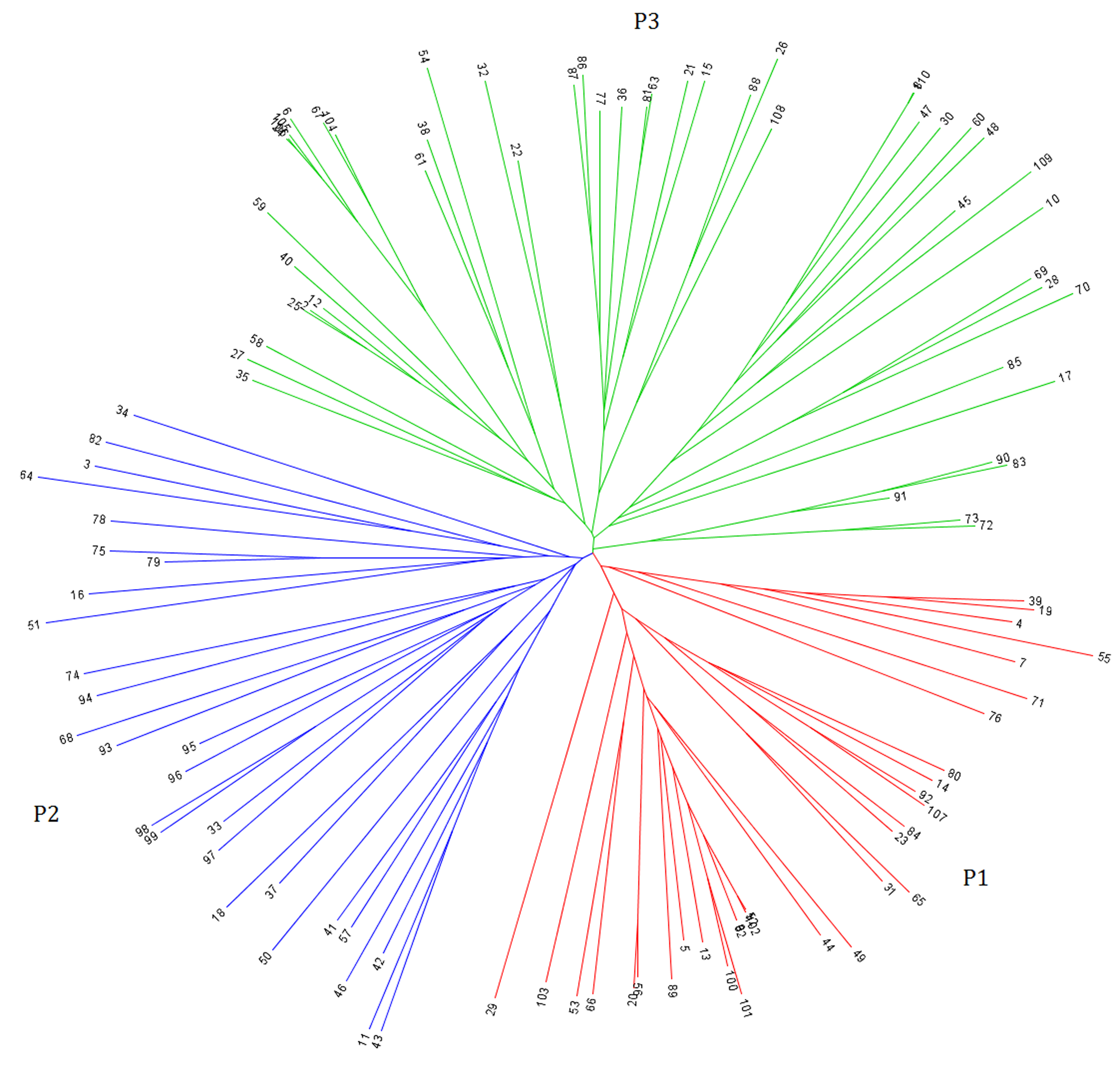

Supplement: S4 Fig — (TIF) [file pone.0142585.s004.tif]

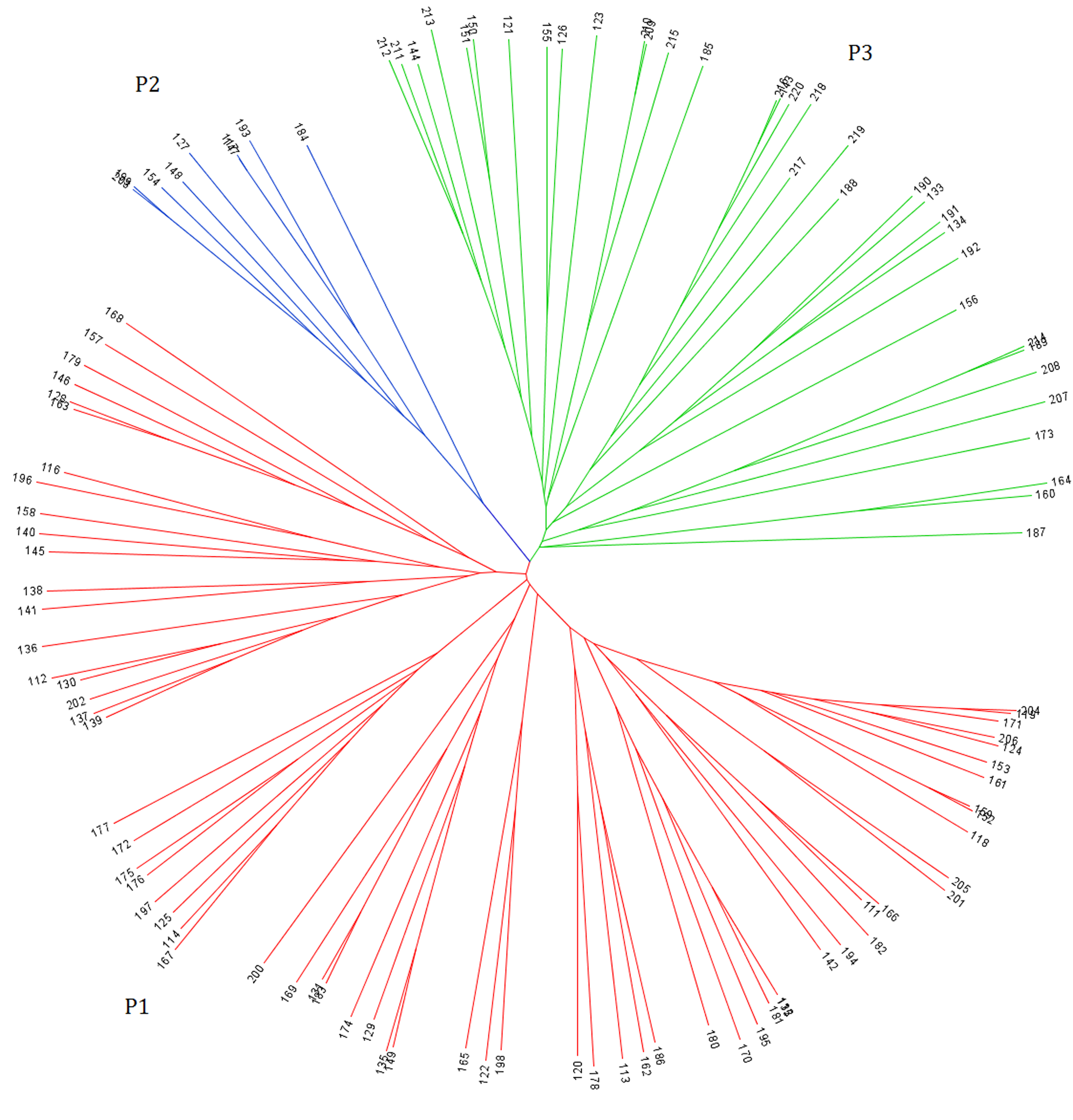

Supplement: S5 Fig — (TIF) [file pone.0142585.s005.tif]
