# Supplementary material for: Identification of Genetic Differentiation between Waxy and Common Maize by SNP Genotyping
Source: PLoS One. 2015 Nov 13;10(11):e0142585. doi: 10.1371/journal.pone.0142585 (PMC4643885; doi:10.1371/journal.pone.0142585)
Supplement: S1 Table — Information provided in this supplemental table includes inbred line or landrace name, and which group the line was assigned to (waxy maize and common maize). (DOCX) [file pone.0142585.s006.docx]

**S1 Table. List of the germplasms used in the study.**

| No. | Inbred line/Landrace | Group |
| --- | --- | --- |
| 1 | Hu-2 | waxy maize |
| 2 | hengbai522 | waxy maize |
| 3 | ZFF | waxy maize |
| 4 | W18 | waxy maize |
| 5 | Xinghongnuo×T5 | waxy maize |
| 6 | 06X—6 | waxy maize |
| 7 | W6 | waxy maize |
| 8 | RF | waxy maize |
| 9 | Tongxi5 | waxy maize |
| 10 | R⑧ | waxy maize |
| 11 | T2 | waxy maize |
| 12 | H11 | waxy maize |
| 13 | NFW5W5 | waxy maize |
| 14 | HMZW | waxy maize |
| 15 | SZW | waxy maize |
| 16 | YHuF | waxy maize |
| 17 | YHeF | waxy maize |
| 18 | T4 | waxy maize |
| 19 | T361 | waxy maize |
| 20 | T354 | waxy maize |
| 21 | Xiangnuo618 | waxy maize |
| 22 | H13 | waxy maize |
| 23 | NF | waxy maize |
| 24 | H9 | waxy maize |
| 25 | YBW1 | waxy maize |
| 26 | HDWF | waxy maize |
| 27 | HLZ | waxy maize |
| 28 | L8 | waxy maize |
| 29 | ZJM | waxy maize |
| 30 | 08M | waxy maize |
| 31 | JNHWF | waxy maize |
| 32 | Huangnian | waxy maize |
| 33 | Zengxiongnuo | waxy maize |
| 34 | Bainian | waxy maize |
| 35 | Shennongnuo | waxy maize |
| 36 | Zhongnuo2M | waxy maize |
| 37 | H·T4 | waxy maize |
| 38 | H366·T4 | waxy maize |
| 39 | W361·T2 | waxy maize |
| 40 | T2·H | waxy maize |
| 41 | T2·H366 | waxy maize |
| 42 | W354·T2 | waxy maize |
| 43 | Huangnian·T2 | waxy maize |
| 44 | T2·Hu-2 | waxy maize |
| 45 | J2000 | waxy maize |
| 46 | H366·T2·T2 | waxy maize |
| 47 | RF·Xiangnuo618 | waxy maize |
| 48 | RF·H9 | waxy maize |
| 49 | Hu-2·RF | waxy maize |
| 50 | Hainannuo | waxy maize |
| 51 | WH8 | waxy maize |
| 52 | W5V | waxy maize |
| 53 | W5·T2 | waxy maize |
| 54 | E77·H366 | waxy maize |
| 55 | Zhenheinuo1·361 | waxy maize |
| 56 | NF354354354 | waxy maize |
| 57 | HW·T2 | waxy maize |
| 58 | Wannian | waxy maize |
| 59 | H·D340 | waxy maize |
| 60 | Hu-1·RF | waxy maize |
| 61 | H366 | waxy maize |
| 62 | R③ | waxy maize |
| 63 | J2M | waxy maize |
| 64 | ZJF | waxy maize |
| 65 | Guoqunuo | waxy maize |
| 66 | T5·T1 | waxy maize |
| 67 | SW1 | waxy maize |
| 68 | L1M | waxy maize |
| 69 | JN2-8 | waxy maize |
| 70 | JN2-9 | waxy maize |
| 71 | Nuo6-2 | waxy maize |
| 72 | H6-1zi | waxy maize |
| 73 | HE6-1 | waxy maize |
| 74 | N7-2 | waxy maize |
| 75 | HE11-1 | waxy maize |
| 76 | H-146 | waxy maize |
| 77 | Q96-2 | waxy maize |
| 78 | hw1401 | waxy maize |
| 79 | Jinghuangnuo | waxy maize |
| 80 | Rudonghuangnuo | waxy maize |
| 81 | 11HLW-3 | waxy maize |
| 82 | 11HC-5 | waxy maize |
| 83 | 11HW-2 | waxy maize |
| 84 | ZFM | waxy maize |
| 85 | HZHW | waxy maize |
| 86 | Ke1-8 | waxy maize |
| 87 | JDWM | waxy maize |
| 88 | HDWM | waxy maize |
| 89 | L150 | waxy maize |
| 90 | NM | waxy maize |
| 91 | SW4 | waxy maize |
| 92 | NTZW | waxy maize |
| 93 | L1F | waxy maize |
| 94 | L8F | waxy maize |
| 95 | Yishannuo | waxy maize |
| 96 | Huangjiangnuo | waxy maize |
| 97 | zhenxiongnuo1 | waxy maize |
| 98 | zhenxiongnuo2 | waxy maize |
| 99 | zhenxiongnuo3 | waxy maize |
| 100 | Tgai | waxy maize |
| 101 | TZ228TZ | waxy maize |
| 102 | TZ359 | waxy maize |
| 103 | TZNongzi | waxy maize |
| 104 | W314HZ | waxy maize |
| 105 | W314228 | waxy maize |
| 106 | W313.3WX | waxy maize |
| 107 | DFHW | waxy maize |
| 108 | Yuheinuo | waxy maize |
| 109 | J2FA | waxy maize |
| 110 | SMLRF | waxy maize |
| 111 | 43.7 | common maize |
| 112 | T458 | common maize |
| 113 | T75 | common maize |
| 114 | 4S | common maize |
| 115 | T877 | common maize |
| 116 | S51M | common maize |
| 117 | C72 | common maize |
| 118 | Nongda1145 | common maize |
| 119 | Qi319 | common maize |
| 120 | T249 | common maize |
| 121 | 9409F | common maize |
| 122 | Dan598 | common maize |
| 123 | 40⑤ | common maize |
| 124 | Shen137 | common maize |
| 125 | 4A·YC | common maize |
| 126 | S51F | common maize |
| 127 | Wu314 | common maize |
| 128 | T812 | common maize |
| 129 | 22M | common maize |
| 130 | X19M | common maize |
| 131 | Zheng58 | common maize |
| 132 | 78599 | common maize |
| 133 | N18 | common maize |
| 134 | N21 | common maize |
| 135 | Dan340 | common maize |
| 136 | K22 | common maize |
| 137 | T803 | common maize |
| 138 | C8605 | common maize |
| 139 | 478 | common maize |
| 140 | SMLYC | common maize |
| 141 | 7922 | common maize |
| 142 | DH02 | common maize |
| 143 | Mo17 | common maize |
| 144 | Zi330 | common maize |
| 145 | DH65232 | common maize |
| 146 | 8723 | common maize |
| 147 | Xun92-6 | common maize |
| 148 | lx9801 | common maize |
| 149 | V14 | common maize |
| 150 | Zong3 | common maize |
| 151 | Zong31 | common maize |
| 152 | T178 | common maize |
| 153 | P138 | common maize |
| 154 | Huangzaosi | common maize |
| 155 | Luyuan92 | common maize |
| 156 | Ye107 | common maize |
| 157 | DH4866 | common maize |
| 158 | JH3372 | common maize |
| 159 | JH78-2 | common maize |
| 160 | Zheng39 | common maize |
| 161 | J-2 | common maize |
| 162 | “66” | common maize |
| 163 | 568G | common maize |
| 164 | H04—24 | common maize |
| 165 | Dongdan60 | common maize |
| 166 | 43.7(249+…) | common maize |
| 167 | S4·4S | common maize |
| 168 | XianfengX | common maize |
| 169 | 4S·Z58 | common maize |
| 170 | Qi319·X7 | common maize |
| 171 | Qi319·78599 | common maize |
| 172 | 137·4S | common maize |
| 173 | XY335 | common maize |
| 174 | E28·CML·Dan340 | common maize |
| 175 | E28·CML·4S | common maize |
| 176 | K12·MDR·Dan340 | common maize |
| 177 | K12·MDR·4S | common maize |
| 178 | 249CML326 | common maize |
| 179 | 568GS3568G | common maize |
| 180 | Zong3·877 | common maize |
| 181 | T249·7 | common maize |
| 182 | T43.7·C72 | common maize |
| 183 | A2M | common maize |
| 184 | LY13M | common maize |
| 185 | D805 | common maize |
| 186 | T53 | common maize |
| 187 | S4 | common maize |
| 188 | S187 | common maize |
| 189 | N9 | common maize |
| 190 | N16 | common maize |
| 191 | N23 | common maize |
| 192 | N24 | common maize |
| 193 | Y85·C72 | common maize |
| 194 | T75·T178 | common maize |
| 195 | E7.7·9045 | common maize |
| 196 | Zong31·S951 | common maize |
| 197 | 4S·C72 | common maize |
| 198 | 4S·Dan598 | common maize |
| 199 | Ji853 | common maize |
| 200 | XG478 | common maize |
| 201 | S651 | common maize |
| 202 | XD20M | common maize |
| 203 | A19 | common maize |
| 204 | CM | common maize |
| 205 | JS045-1 | common maize |
| 206 | JS06730 | common maize |
| 207 | BSSS(R)C7 | common maize |
| 208 | BS13(S)C5 | common maize |
| 209 | (PA91/LH98)GA-6-420-64-2-1-3-3-1 | common maize |
| 210 | (PA91/LH98A)-6-420-64-2-1-1-5-1-1 | common maize |
| 211 | A619×L120 | common maize |
| 212 | Oh43×L120 | common maize |
| 213 | LH19×LH39 | common maize |
| 214 | B73(2)×H93 | common maize |
| 215 | Va85×Pa91 | common maize |
| 216 | Mo17 backcross 5 recovery | common maize |
| 217 | Mo17(3)×610 (610=W153R type) | common maize |
| 218 | [(Mo17×H99)LH53] | common maize |
| 219 | LH55×LH47 | common maize |
| 220 | [(Mo17×ASA)Mo17(2)] | common maize |
