# Supplementary material for: Identification of Genetic Differentiation between Waxy and Common Maize by SNP Genotyping
Source: PLoS One. 2015 Nov 13;10(11):e0142585. doi: 10.1371/journal.pone.0142585 (PMC4643885; doi:10.1371/journal.pone.0142585)
Supplement: S2 Table — (DOCX) [file pone.0142585.s007.docx]

**S2 Table** Genes and SNPs identified within the distinct LD block on chromosome 6 and chromosome 8.

| **SNPs in distinct LD block** | **Chr.** | **Position** | **Genes in the LD block** | **Annotation** | **Putative functions** |
| --- | --- | --- | --- | --- | --- |
| PZE-106078719 | 6 | 134460941 | GRMZM2G424783  GRMZM2G329033  GRMZM2G357198  GRMZM2G055678  GRMZM2G100067  GRMZM2G061469  GRMZM2G162702  GRMZM2G170646  GRMZM2G462625  GRMZM2G531738 | Emp24 family protein  Transcription factor IIA  Transposable element gene  Proline extensin-like receptor kinase 1  RAD3-like DNA-binding helicase protein  SLAC1 homologue 3  Serine/threonine-protein kinase  GDSL-like lipase/acylhydrolase  Tetratricopeptide repeat (TPR)-like protein  MYB family transcription factor | Response to heat-shock and cell death[[1](#_ENREF_1)]  Activated transcription[[2](#_ENREF_2)]  Defense responses[[3](#_ENREF_3)]  DNA Repair[[4](#_ENREF_4)]  Stomatal signalling[[5](#_ENREF_5)]  Strss response[[6](#_ENREF_6)]  Responses to biotic and abiotic stresses[[7](#_ENREF_7)]  Modulates development[[8](#_ENREF_8)]  Strss response and development control[[9](#_ENREF_9)] |
| PZE-106078723 | 6 | 134461413 |  |  |  |
| PZE-106078726 | 6 | 134461547 |  |  |  |
| PZE-106078845 | 6 | 134651274 |  |  |  |
| PZE-106078910 | 6 | 134657125 |  |  |  |
| PZE-106078990 | 6 | 134846181 |  |  |  |
| PZE-106079085 | 6 | 134863316 |  |  |  |
| SYN35781 | 6 | 134904332 |  |  |  |
| PZE-106079198 | 6 | 135128415 |  |  |  |
| SYN36674 | 6 | 135143457 |  |  |  |
| PZE-108035543 | 8 | 52204797 | GRMZM2G477457  AC205274.3_FG001  GRMZM2G047966  GRMZM2G047998  GRMZM5G814722  GRMZM2G107718  AC235535.1_FG001  GRMZM2G017666  GRMZM2G439589  GRMZM2G302405  GRMZM5G850758  GRMZM2G180488  GRMZM2G173119  GRMZM2G015959  GRMZM2G384871  GRMZM2G131074  GRMZM2G094808  GRMZM6G514393  AC194355.3_FG002  GRMZM2G001755 | K^+^ uptake transporter 3  Pathogenesis-related gene 1  Transposable element gene  Transposable element gene  Transposable element gene  Ribosomal protein L5  Chromatin-remodeling protein  Hypothetical protein  NAD(P)-binding protein  TPR-like superfamily protein  Hypothetical protein  Hypothetical protein  Associated molecule with SH3 of STAM 3  DegP protease 9  Phosphoenolpyruvate carboxykinase  Hypothetical protein  Hypothetical protein  Peroxidase superfamily protein  mTERF family protein  Hypothetical protein | Compete for nutrients[[10](#_ENREF_10)]  Systemic resistance[[11](#_ENREF_11)]  Ribosomal biogenesis stress[[12](#_ENREF_12)]  Responses to stresses and plant development[[13](#_ENREF_13)]  Disease resistance[[14](#_ENREF_14)]  Modulate development[[8](#_ENREF_8)]  Intracellular trafficking and vacuole biogenesis[[15](#_ENREF_15)]  Thermal and oxidative tolerance[[16](#_ENREF_16)]  Photosynthesis[[17](#_ENREF_17)]  Plant defense and cell wall metabolism[[18](#_ENREF_18)]  Mitochondrion, chloroplast and leaf development[[19](#_ENREF_19)] |
| PZE-108035545 | 8 | 52205440 |  |  |  |
| PZA00498.5 | 8 | 52299429 |  |  |  |
| SYN25157 | 8 | 52299429 |  |  |  |
| PZE-108035582 | 8 | 52299792 |  |  |  |
| PZE-108035583 | 8 | 52299869 |  |  |  |
| PZE-108035671 | 8 | 52902989 |  |  |  |
| PZE-108035778 | 8 | 53329919 |  |  |  |
| SYN34307 | 8 | 53872820 |  |  |  |
| PZE-108035902 | 8 | 53874805 |  |  |  |
| PZE-108035926 | 8 | 53955319 |  |  |  |
| PZE-108035929 | 8 | 53957102 |  |  |  |
| PZE-108035931 | 8 | 53957272 |  |  |  |
| PZE-108036026 | 8 | 54324318 |  |  |  |
| PZE-108036054 | 8 | 54428423 |  |  |  |
| PZE-108036058 | 8 | 54444675 |  |  |  |
| PZE-108036077 | 8 | 54477661 |  |  |  |
| PZE-108036152 | 8 | 54888683 |  |  |  |
| PZE-108036175 | 8 | 55020791 |  |  |  |
| PZE-108036208 | 8 | 55169464 |  |  |  |
| PZE-108036220 | 8 | 55251243 |  |  |  |
| PZE-108036232 | 8 | 55259181 |  |  |  |
| PZE-108036270 | 8 | 55516140 |  |  |  |
| PZE-108036386 | 8 | 55966495 |  |  |  |
| PZE-108036444 | 8 | 56167353 |  |  |  |
| PZA01209.1 | 8 | 56203327 |  |  |  |

**References:**

1. Hwang SO, Boswell SA, Seo J-S, Lee SW (2008) Novel oxidative stress-responsive gene ERS25 functions as a regulator of the heat-shock and cell death response. Journal of Biological Chemistry 283: 13063-13069.

2. Ma D, Watanabe H, Mermelstein F, Admon A, Oguri K, et al. (1993) Isolation of a cDNA encoding the largest subunit of TFIIA reveals functions important for activated transcription. Genes & Development 7: 2246-2257.

3. Silva N, Goring D (2002) The proline-rich, extensin-like receptor kinase-1 (PERK1) gene is rapidly induced by wounding. Plant Molecular Biology 50: 667-685.

4. Jones NJ, Strike P (1996) Recent research in DNA repair, mutation and recombination: A report of the DNA Repair Network meeting, held at City University, London on 18 December 1995. Mutation Research/DNA Repair 364: 13-23.

5. Vahisalu T, Kollist H, Wang Y-F, Nishimura N, Chan W-Y, et al. (2008) SLAC1 is required for plant guard cell S-type anion channel function in stomatal signalling. Nature 452: 487-491.

6. Sun X-L, Yu Q-Y, Tang L-L, Ji W, Bai X, et al. (2013) GsSRK, a G-type lectin S-receptor-like serine/threonine protein kinase, is a positive regulator of plant tolerance to salt stress. Journal of plant physiology 170: 505-515.

7. Wang W, Zhao X, Pan Y, Zhu L, Fu B, et al. (2011) DNA methylation changes detected by methylation-sensitive amplified polymorphism in two contrasting rice genotypes under salt stress. Journal of Genetics and Genomics 38: 419-424.

8. Lin Z, Ho C-W, Grierson D (2009) AtTRP1 encodes a novel TPR protein that interacts with the ethylene receptor ERS1 and modulates development in Arabidopsis. Journal of experimental botany: erp209.

9. Du H, Feng B-R, Yang S-S, Huang Y-B, Tang Y-X (2012) The R2R3-MYB transcription factor gene family in maize. PloS one 7: e37463.

10. Ashley M, Grant M, Grabov A (2006) Plant responses to potassium deficiencies: a role for potassium transport proteins. Journal of experimental botany 57: 425-436.

11. Han B, Chen L, Wang J, Wu Z, Yan L, et al. (2015) Constitutive Expresser of Pathogenesis Related Genes 1 Is Required for Pavement Cell Morphogenesis in Arabidopsis. PloS one 10: e0133249.

12. Bursać S, Brdovčak MC, Pfannkuchen M, Orsolić I, Golomb L, et al. (2012) Mutual protection of ribosomal proteins L5 and L11 from degradation is essential for p53 activation upon ribosomal biogenesis stress. Proceedings of the National Academy of Sciences 109: 20467-20472.

13. Reyes JC, Hennig L, Gruissem W (2002) Chromatin-remodeling and memory factors. New regulators of plant development. Plant physiology 130: 1090-1101.

14. Dehury B, Patra MC, Maharana J, Sahu J, Sen P, et al. (2014) Structure-based computational study of two disease resistance gene homologues (Hm1 and Hm2) in maize (Zea mays L.) with implications in plant-pathogen interactions.

15. Isono E, Katsiarimpa A, Müller IK, Anzenberger F, Stierhof Y-D, et al. (2010) The deubiquitinating enzyme AMSH3 is required for intracellular trafficking and vacuole biogenesis in Arabidopsis thaliana. The Plant Cell 22: 1826-1837.

16. Jones CH, Tove'C B, Jones KF, Zeller GO, Hruby DE (2001) Conserved DegP Protease in Gram-Positive Bacteria Is Essential for Thermal and Oxidative Tolerance and Full Virulence inStreptococcus pyogenes. Infection and Immunity 69: 5538-5545.

17. Leegood RC, Walker RP (2003) Regulation and roles of phosphoenolpyruvate carboxykinase in plants. Archives Of Biochemistry And Biophysics 414: 204-210.

18. O’Brien JA, Daudi A, Butt VS, Bolwell GP (2012) Reactive oxygen species and their role in plant defence and cell wall metabolism. Planta 236: 765-779.

19. Quesada V, Sarmiento‐Mañús R, González‐Bayón R, Hricová A, Pérez‐Marcos R, et al. (2011) Arabidopsis RUGOSA2 encodes an mTERF family member required for mitochondrion, chloroplast and leaf development. The Plant Journal 68: 738-753.
