# Supplementary material for: Identification of Genetic Differentiation between Waxy and Common Maize by SNP Genotyping
Source: PLoS One. 2015 Nov 13;10(11):e0142585. doi: 10.1371/journal.pone.0142585 (PMC4643885; doi:10.1371/journal.pone.0142585)

**S1 File** LD comparasion within all chromosomes between waxy and maize groups

Chr.1 of waxy maize

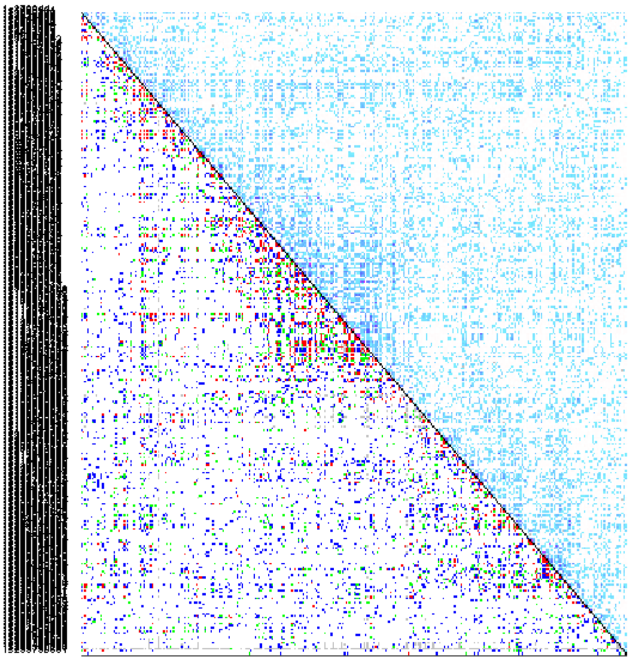

Chr.1 of common maize

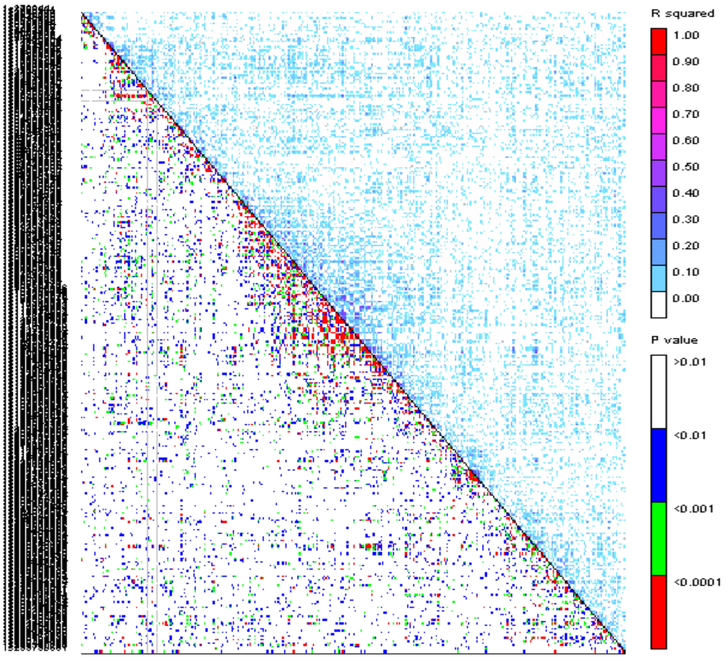


Chr.2 of waxy maize

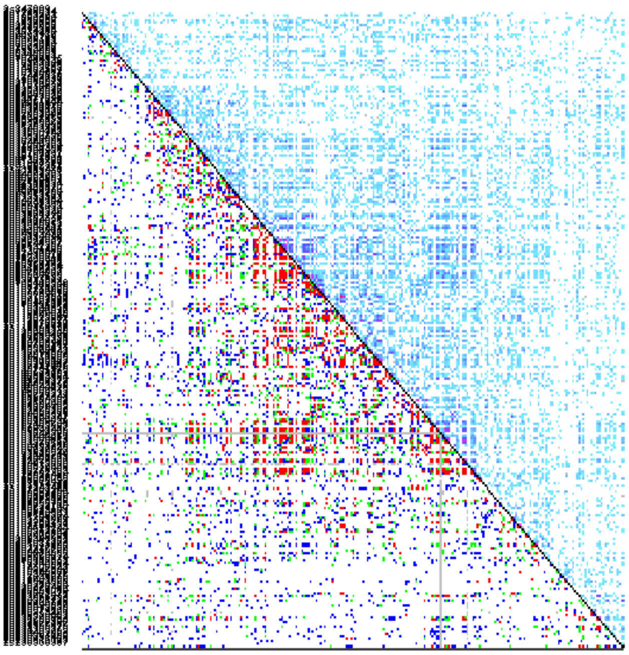

Chr.2 of common maize

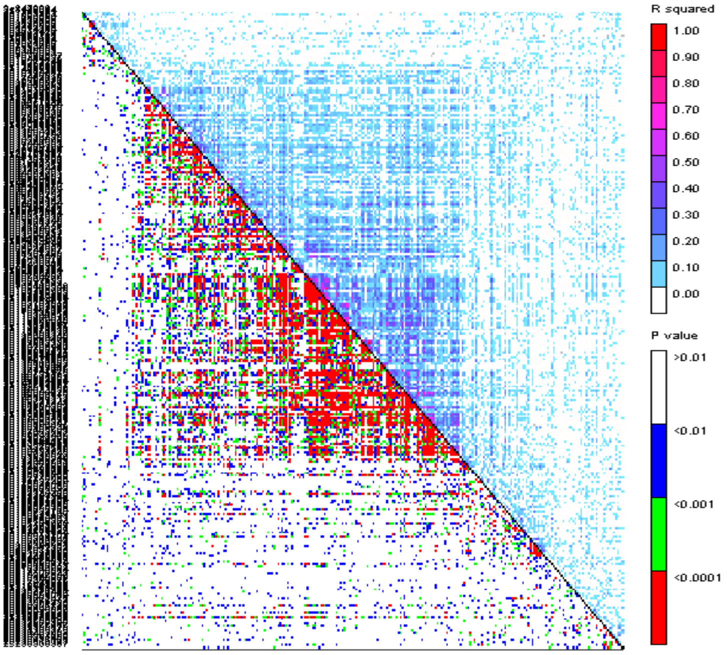

Chr.3 of waxy maize

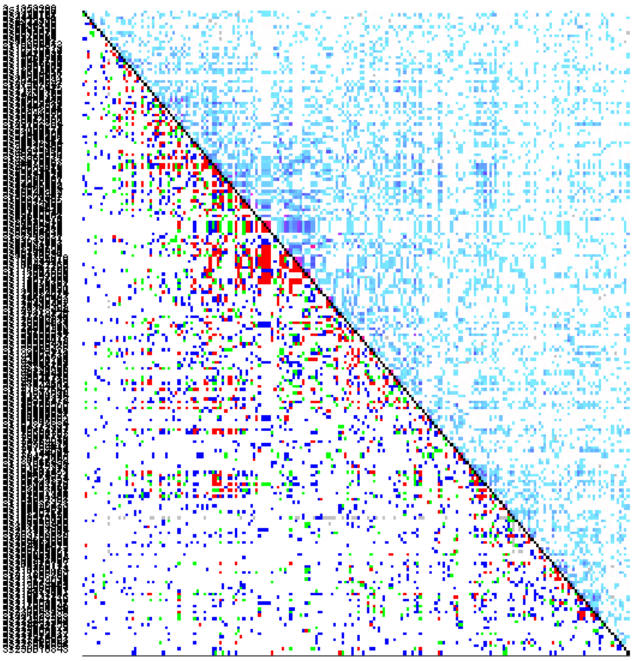

Chr.3 of common maize

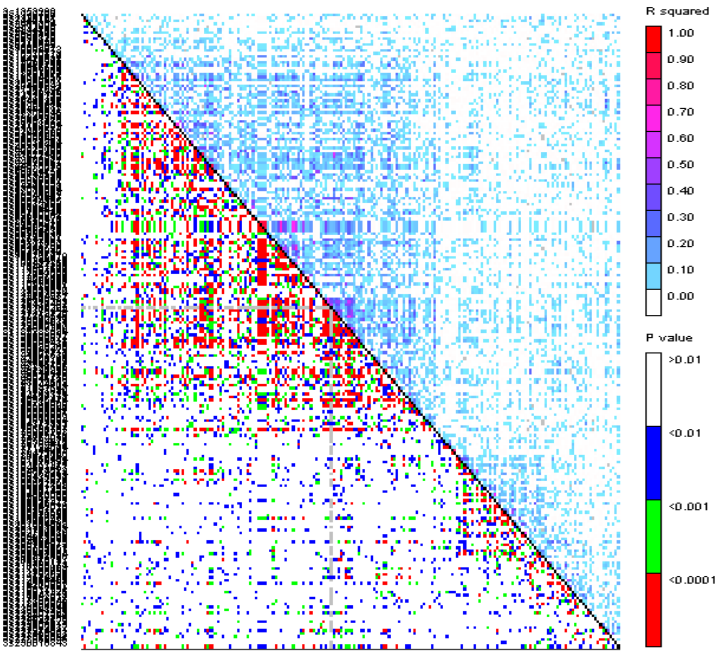


Chr.4 of waxy maize

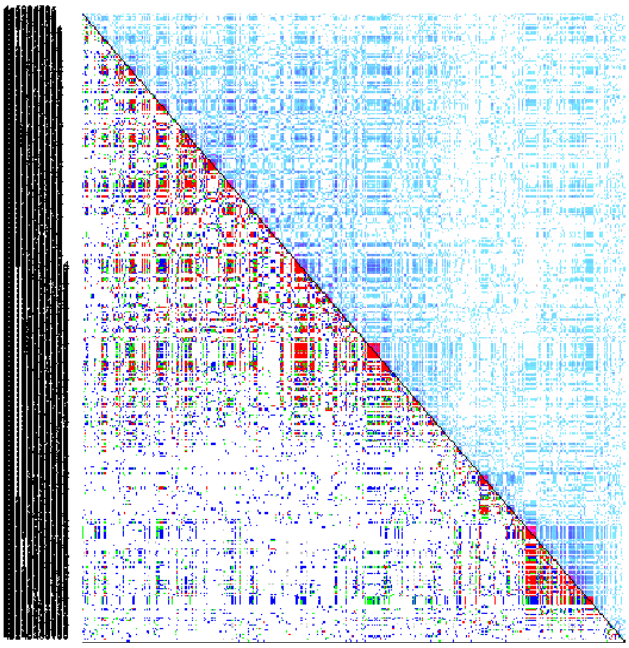

Chr.4 of common maize

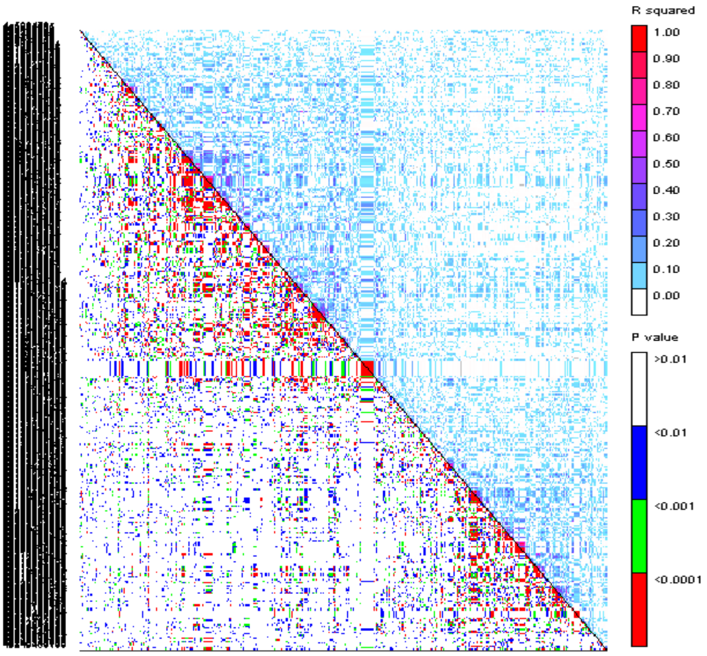

Chr.5 of waxy maize

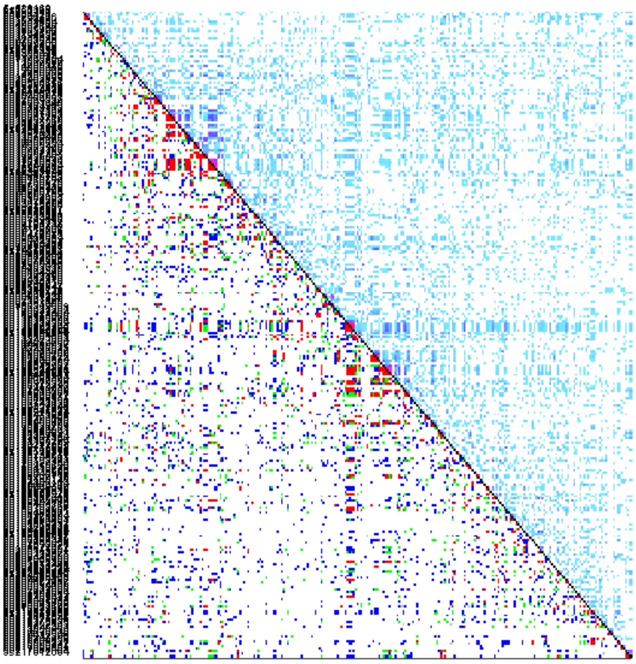

Chr.5 of common maize

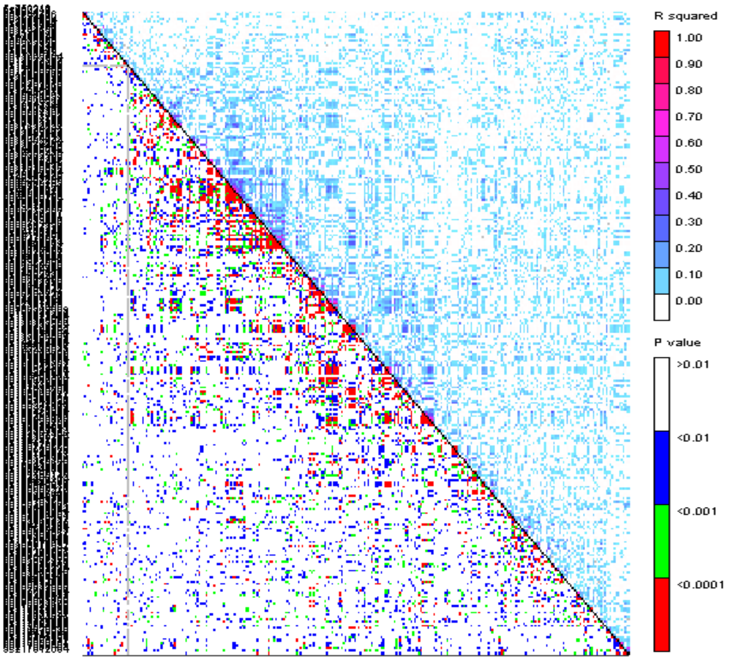


Chr.6 of waxy maize

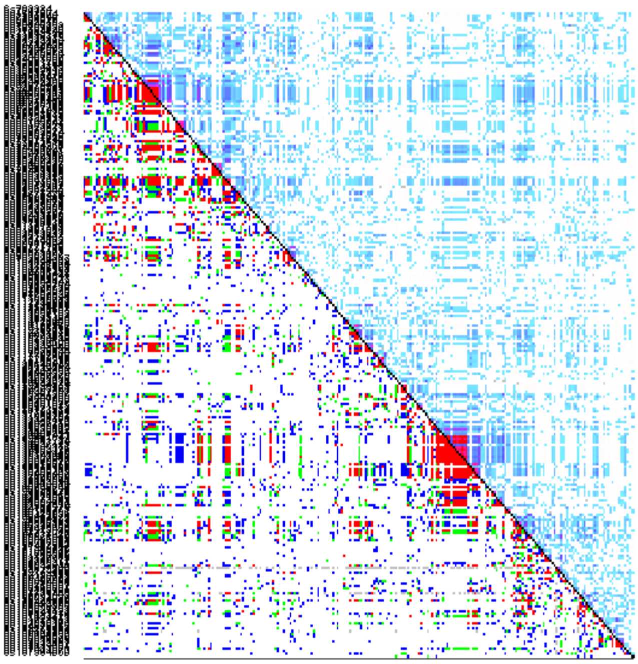

Chr.6 of common maize

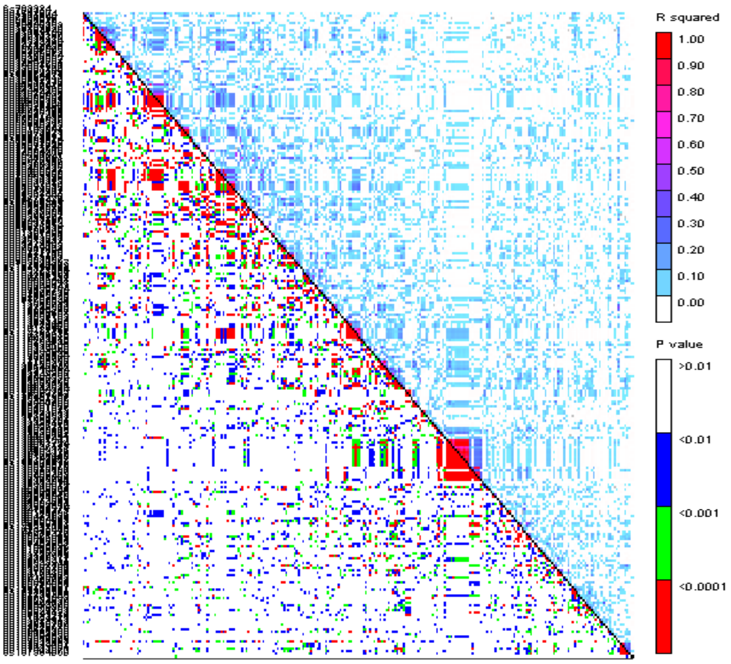

Chr.7 of waxy maize

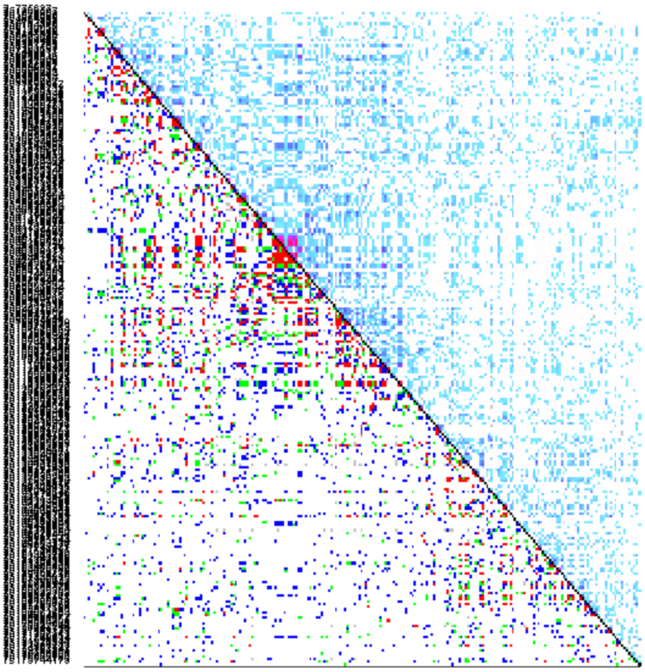

Chr.7 of common maize

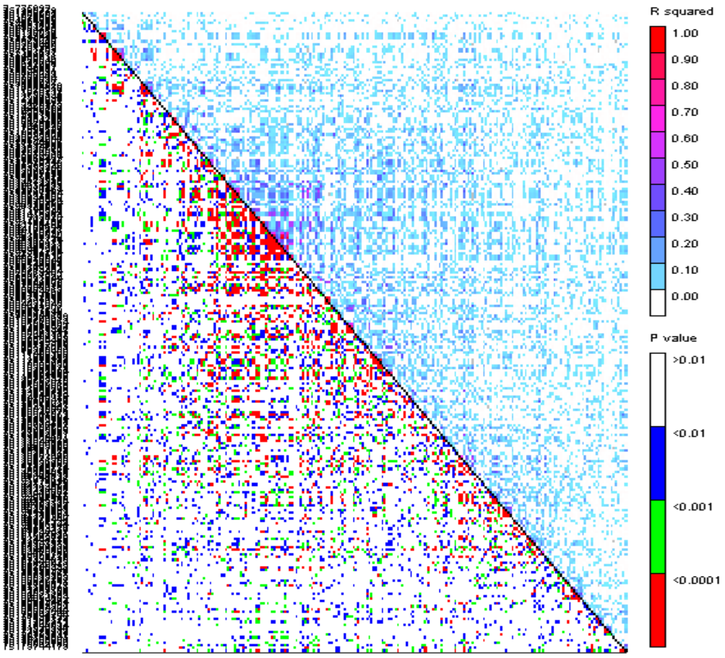


Chr.8 of waxy maize

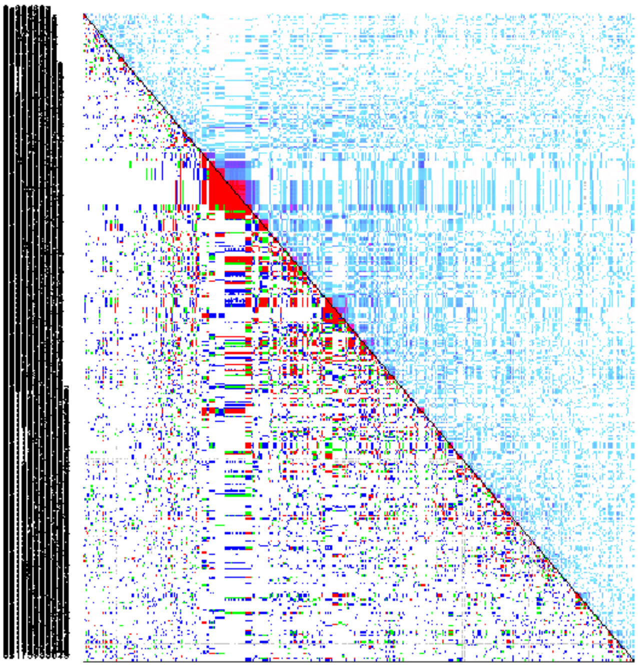

Chr.8 of common maize

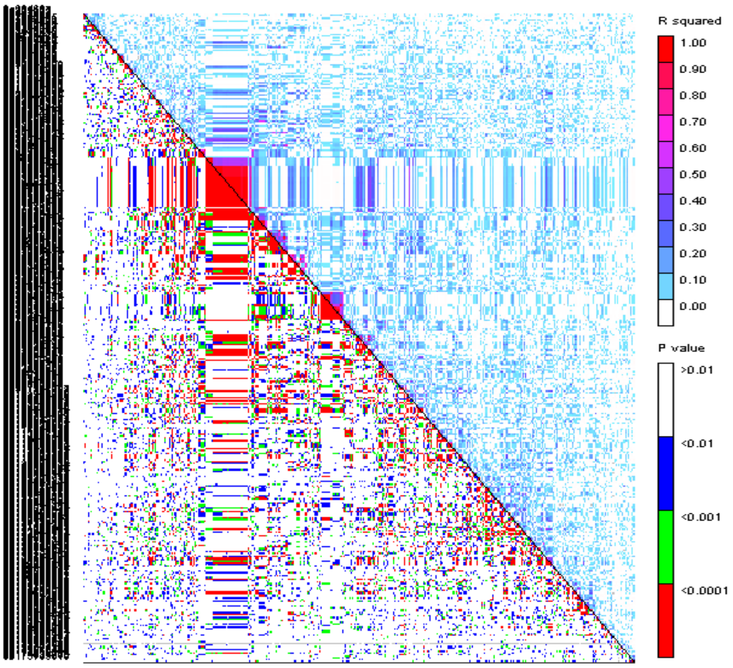

Chr.9 of waxy maize

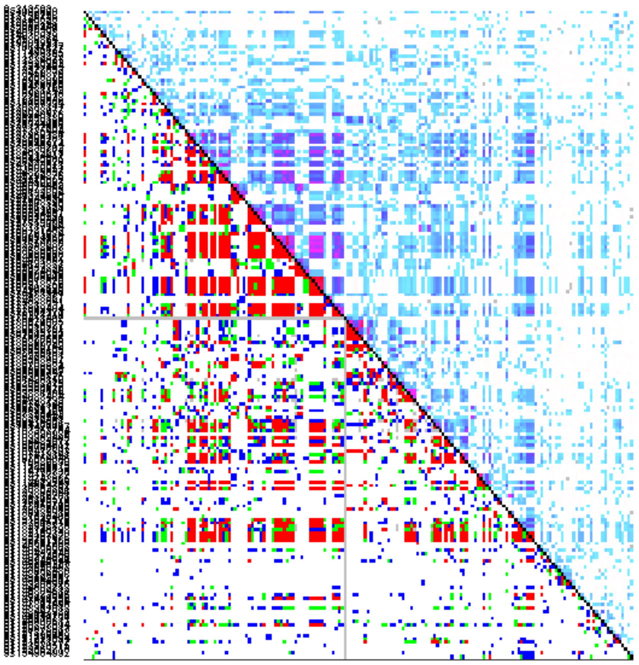

Chr.9 of common maize

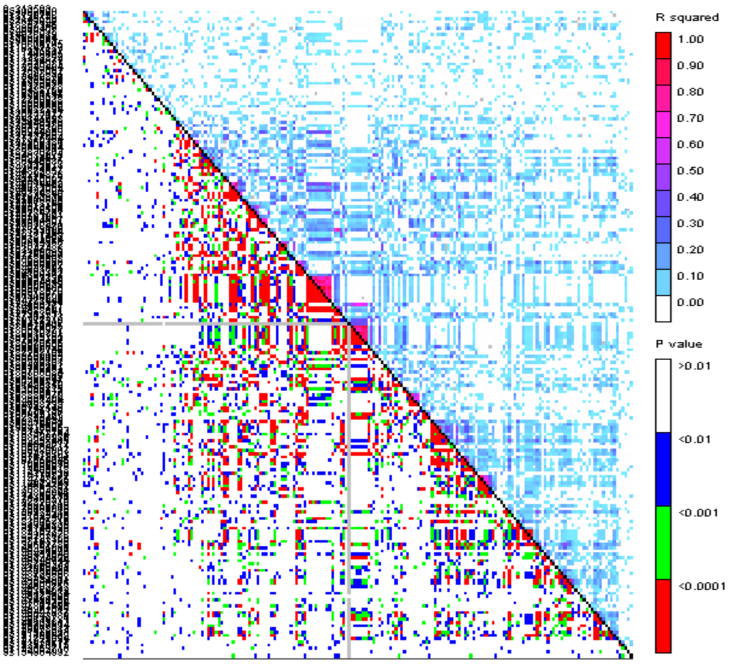


Chr.10 of waxy maize

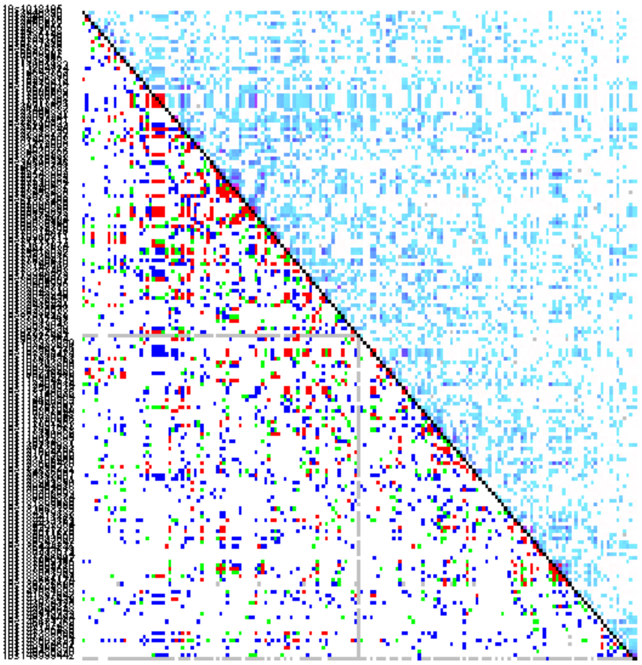

Chr.10 of common maize

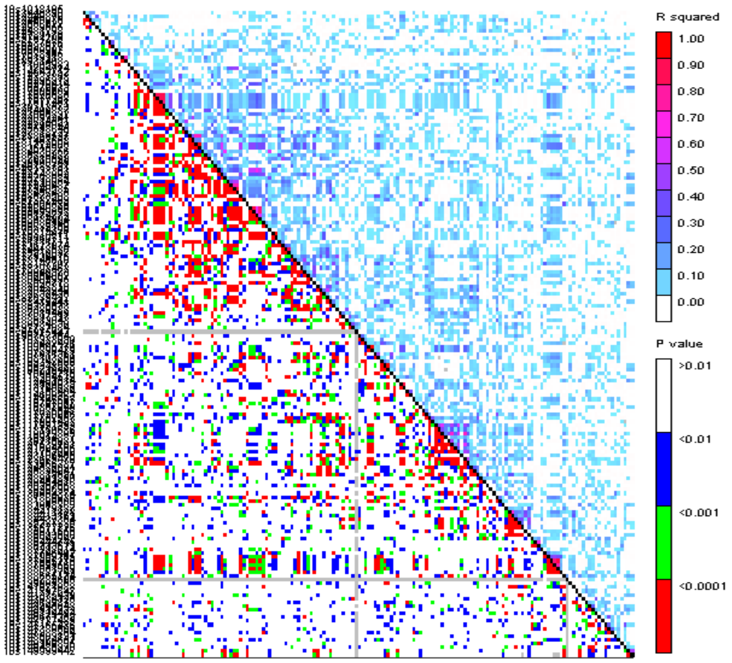

Supplement: S1 File — (PDF) [file pone.0142585.s008.pdf]
